# Clinical characteristics of *Demodex*-associated recurrent hordeola: an observational, comparative study

**DOI:** 10.1038/s41598-021-00599-7

**Published:** 2021-11-01

**Authors:** Sung Yeon Jun, Yeon Jung Choi, Bo Ram Lee, Sang Un Lee, Sung Chul Kim

**Affiliations:** 1 HanGil Eye Hospital, 35, Bupyeong-daero, Bupyeong-gu, Incheon, 21388 South Korea; 2 Department of Ophthalmology, College of Medicine, Catholic Kwandong University, Gangneung, South Korea

**Keywords:** Health care, Medical research, Risk factors, Signs and symptoms

## Abstract

Our study evaluated the association between *Demodex* infestation and recurrent hordeola and examined the clinical features associated with these eyelid lesions. This was an observational, comparative study. We reviewed 250 patients and divided them into the recurrent hordeolum (n = 153) and control (n = 97) groups. *Demodex* infestation was detected by epilating eyelashes around the lesion/s and viewing them under a light microscope. Patient medical records and photographs were retrospectively analyzed to identify the clinical characteristics of *Demodex*-associated recurrent hordeola. *Demodex* was detected in 91 (59.5%) and 17 (17.5%) patients in the recurrent hordeolum and control groups (*p* < 0.001), respectively. In the recurrent hordeolum group, *Demodex* mites were found in 74 (68.5%) and 17 (37.8%) of the adult and pediatric patients (*p* < 0.001), respectively. Among patients with recurrent hordeola, patients in their 20s were most likely to have concomitant *Demodex* infestation. Patients with *Demodex* infestations were also more likely to develop recurrent lesions within a shorter period of time from the primary incision and curettage. The most common presentation of *Demodex*-associated recurrent lesions was external hordeola (67%) (*p* = 0.002). *Demodex* infestation may cause recurrent hordeola in adults and children. These mites may play a greater role in the development of lesions in adult patients. The strongest association between *Demodex* infestation and recurrent lesions was seen in patients in their 20s. Our results suggest that if the hordeola recur within a short period of time with the clinical characteristics of external location of eyelid, multiple numbers of lesions, or anterior blepharitis, eyelash epilation should be performed to identify the presence of *Demodex* mites.

## Introduction

*Demodex* mites are normal inhabitants of the pilosebaceous unit and considered part of the normal skin flora^1^. Recent studies have reported that ocular demodicidosis, which is considered be a new type of blepharitis is a potential risk factor for chalazia^2–5^. In particular, recent studies by Yam and Liang reported that *Demodex* infestation is associated with chalazion recurrence^2,3^. However, Liang’s study did not include patients in the acute stage, which may be a limitation in presenting the exact clinical characteristics of chalazion recurrence. Although sufficient medication may have been administered and surgical removal performed, chalazion or hordeolum recurrence in a relatively short period of time is embarrassing for ophthalmologists. However, the treatment of these patients may be relatively easy if ophthalmologists are aware of the clinical features of recurrent *Demodex* infestations*.* The medical and surgical treatment of mite-associated chalazion and/or hordeolum cannot prevent their recurrence; however, tea tree oil (TTO), Terpinen-4-ol, 4% pilocarpine gel, and systemic ivermectin have been advocated as the primary treatment for patients with *Demodex* blepharitis^6,7^. We conducted an observational, comparative study to examine the association between *Demodex* infestations and recurrent hordeola and a retrospective review of the medial records and photographs of patients with recurrent lesions to identify the clinical features of these lesions.

## Methods

**Figure 1:**
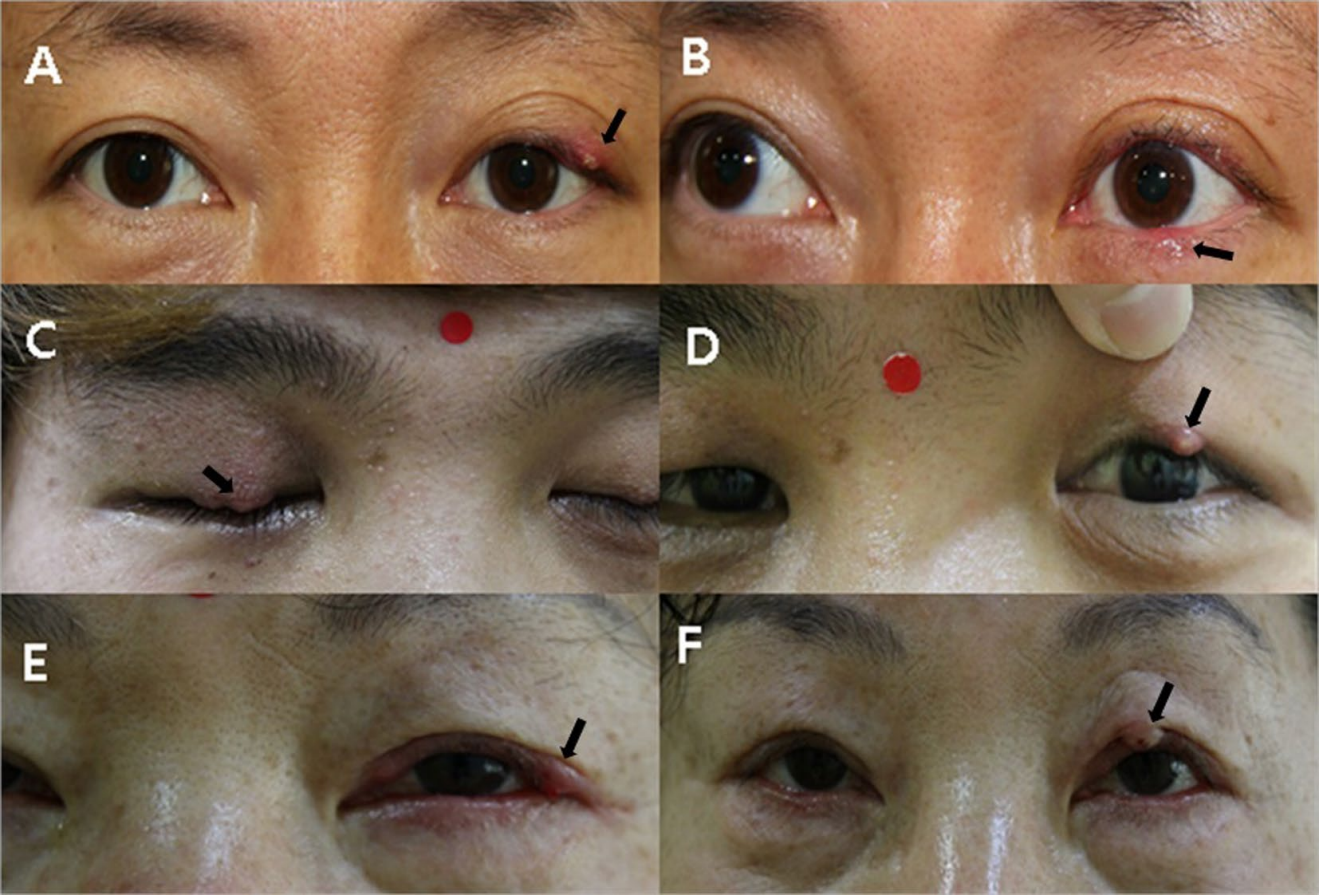
Patient photographs of *Demodex*-associated recurrent hordeola. A 54-year-old man (**A**,**B**), 21-year-old man (**C**,**D**), and 63-year-old-woman (**E**,**F**) demonstrate external hordeola (arrows), which recurred after a short period of time following incision and curettage. All of these patients had *Demodex* mites on the cilia surrounding the lesions.

This was an observational, comparative study. The study followed the tenets of the Declaration of Helsinki and was approved by the Institutional Review Board and Ethics Committee of the HanGil Eye Hospital (HanGil IRB-17008). The same review board waived the requirement for written informed consent, but the representative patients whose photographs were included in this manuscript provided written informed consent (Fig. 1). In total, 250 patients who underwent light microscopic examination to detect *Demodex* infestation between July 2015 to March 2021 were analyzed.

Recurrence was defined as a focal inflamed lesion occurring on the same or another site of the eyelid within 3 months after incision and curettage of the primary lesion. Incision and curettage of the primary lesions was performed by a single surgeon (SC KIM). Postoperatively, all patients received oral antibiotics (amoxicillin hydrate and clavulanate) thrice daily, combined steroid (Fluorometholone 0.1%, Santen Pharmaceutical Korea Co., Ltd.) and antibiotics (Cravit^®^, Levofloxacin 0.5%, Santen Pharmaceutical Korea Co., Ltd.) eyedrops four times daily, and an antibiotic ointment (Tarivid^®^, Ofloxain 0.3%, Santen Pharmaceutical Korea Co., Ltd.) thrice daily for 2 weeks on skin, if transcutaneous incision was done. The group with recurrent lesions (n = 153) were analyzed as adult (≥ 15-years, n = 108) and pediatric (< 15-years, n = 45) patients. We analyzed the lesions according to age because *Demodex* infestations have been demonstrated to increase with age^3^. The control group (n = 97) consisted of patients who showed no evidence of focal inflammation on slit microscopic examination, underwent lid surgeries, such as blepharoplasty, entropion repair, epiblepharon repair, and congenital ptosis repair, and lacrimal procedures, for example, dacryocystorhinostomy for acquired and congenital lacrimal duct obstruction, were included (Table 1). Patients with acute conjunctivitis and keratitis and those taking topical or systemic immunosuppressants when hordeolum recurrence was confirmed were excluded. We also excluded patients with any other types of dermatoses such as rosacea, periocular dermatitis, and discoid lupus erythematosus due to their known strong association with *Demodex* infestation.

**Table 1:** The prevalence of *Demodex* mites in recurrent hordeolum group and control group.

	Total (n = 250)			Adult (n = 174)			Pediatric (n = 76)		
	Recurrent group	Control group	*p*^†^	Recurrent group	Control group	*p*^†^	Recurrent group	Control group	*p*^†^
Total number	153	97		108	66		45	31	
M:F	64:89	37:60	0.222	46:62	22:44	0.222	18:27	15:16	0.468
Mean age (yr)	26.9 ± 19.03 (1–66)	33.49 ± 24.64 (1–75)	0.026*	36.03 ± 14.99 (14–66)	46.82 ± 18.15 (14–75)	< 0.001*	4.98 ± 2.86 (1–13)	4.90 ± 2.73 (1–10)	0.815
*Demodex* infestation (%)	91/153 (59.5%)	17/97 (17.5%)	< 0.001*	74/108 (68.5%)	14/66 (21.2%)	< 0.001*	17/45 (37.8%)	3/31 (9.7%)	0.006*
*Odds ratio*	3.39			3.23			5.66		

Recurrent group: Recurrent hordeolum patient group.

Values are expressed as the mean ± standard deviation.

M = Male, F = Female.

^†^Chi-square test.

*p < 0.05.

Patient medical records and photographs were examined retrospectively to identify the clinical characteristics of recurrent lesions and classified according to the presence of *Demodex* mites (Table 2). We examined the lid margin to check for (1) signs of blepharitis, such as margin hyperemia, scaling, crusting, and margin hypertrophy; (2) differentiating features, such as involvement of the lid margin in external hordeola, involvement of the tarsal plate in internal hordeola, and the presence of firm, painless tarsal plate lesions in chalazia; and (3) location and number of the lesions.

**Table 2:** The clinical characteristics of recurrent hordeolum lesion.

	Total			Adult			Pediatric		
	*Demodex* (+)	*Demodex* (−)	*p*^†^	*Demodex* (+)	*Demodex* (−)	*p*^†^	*Demodex* (+)	*Demodex* (−)	*p*^†^
	N = 91	N = 59		N = 74	N = 34		N = 17	N = 25	
**Clinical appearance**									
External	61 (67.0%)	27 (45.8%)	0.002*	49 (66.2%)	12 (35.2%)	0.001*	12 (70.5%)	15 (60%)	0.408
Internal	21 (23.0%)	28 (47.4%)		17 (22.9%)	19 (55.8%)		4 (23.5%)	9 (36%)	
Mixed	9 (9.9%)	4 (6.8%)		8 (10.8%)	3 (8.8%)		1 (5.8%)	1 (4%)	
**Recurrent site**									
Upper eyelid	52 (57.1%)	36 (61.0%)	0.444	43 (58.1%)	22 (64.7%)	0.195	9 (52.9%)	14 (56%)	0.736
Lower eyelid	30 (32.9%)	15 (25.4%)		26 (35.1%)	7 (20.5%)		4 (23.5%)	8 (32%)	
Mixed	9 (9.9%)	8 (13.6%)		5 (6.7%)	5 (14.7%)		4 (23.5%)	3 (12%)	
**Anterior blepharitis**									
(+)	73 (80.2%)	21 (35.6%)	< 0.001*	59 (79.7%)	9 (26.4%)	< 0.001*	14 (82.3%)	12 (48%)	0.024*
(−)	18 (19.8%)	38 (64.4%)		15 (20.2%)	25 (73.5%)		3 (17.6%)	13 (52%)	
**Number of hordeolum**									
One	59 (64.8%)	50 (84.7%)	0.008*	50 (67.5%)	29 (85.2%)	0.054	9 (52.9%)	21 (84%)	0.029*
More than one	32 (35.15)	9 (15.2%)		24 (32.4%)	5 (14.7%)		8 (47.0%)	4 (16%)	

^†^Chi-square test, *p < 0.05.

**Figure 2:**
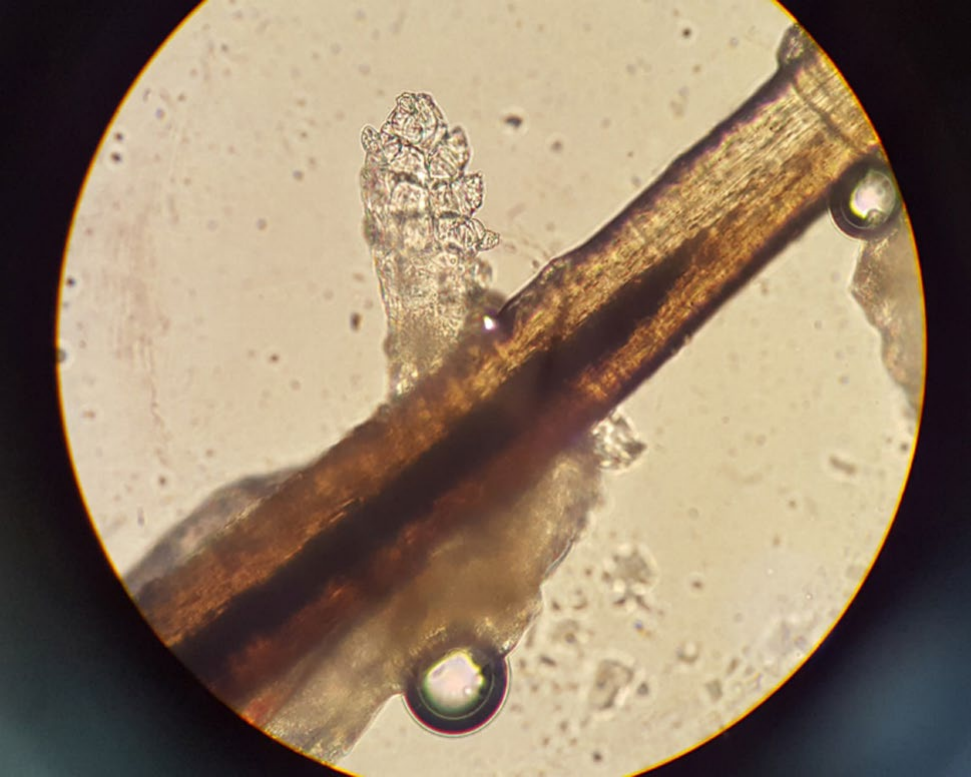
*Demodex* mites on cilia. Microscopic examination demonstrates *Demodex* mites on the cilia.

To evaluate for the presence of *Demodex* mites, we epilated approximately 10 eyelashes around the lesions in the recurrent lesion group. Lash sampling in the pediatric group was performed under general anesthesia prior to surgery. The epilated lashes were placed on a slide glass, moistened with a drop of normal saline, sealed with a cover glass, and observed under a light microscope (Olympus^®^, Tokyo, Japan) (Fig. 2). *Demodex* was considered as present if at least one *Demodex* mite was identified on microscopic examination, whether it was alive or not. Additionally, in the 19 recurrent patients, *Demodex* counting was performed by an independent doctor who had no knowledge of each patient’s clinical information.

Statistical analyses were performed using SPSS for Windows version 25.0 (SPSS Inc., Chicago, Illinois, USA). The chi-square test was used to compare the proportion of *Demodex* mites between adult and pediatric patients in each group. Odds ratios were used to compare between the groups. All values were presented as means ± standard deviations. A *p*-value < 0.05 was considered as statistically significant.

## Results

This study examined 153 patients with recurrent hordeola and 97 patients as controls. The recurrent hordeolum group included 108 and 45 adult and pediatric patients, respectively, whereas the control group included 66 and 31 adult and pediatric patients, respectively. In the recurrent hordeolum group, the mean ages of the adult and pediatric patients were 36.03 ± 14.99-years (range, 14–66 years) and 4.98 ± 2.86 years (range, 1–13 years), respectively. In the control group, the mean ages of the adult and pediatric patients were 46.82 ± 18.15-years (range, 14–75 years) and 4.90 ± 2.73-years (range, 1–10 years), respectively (Table 1).

**Figure 3:**
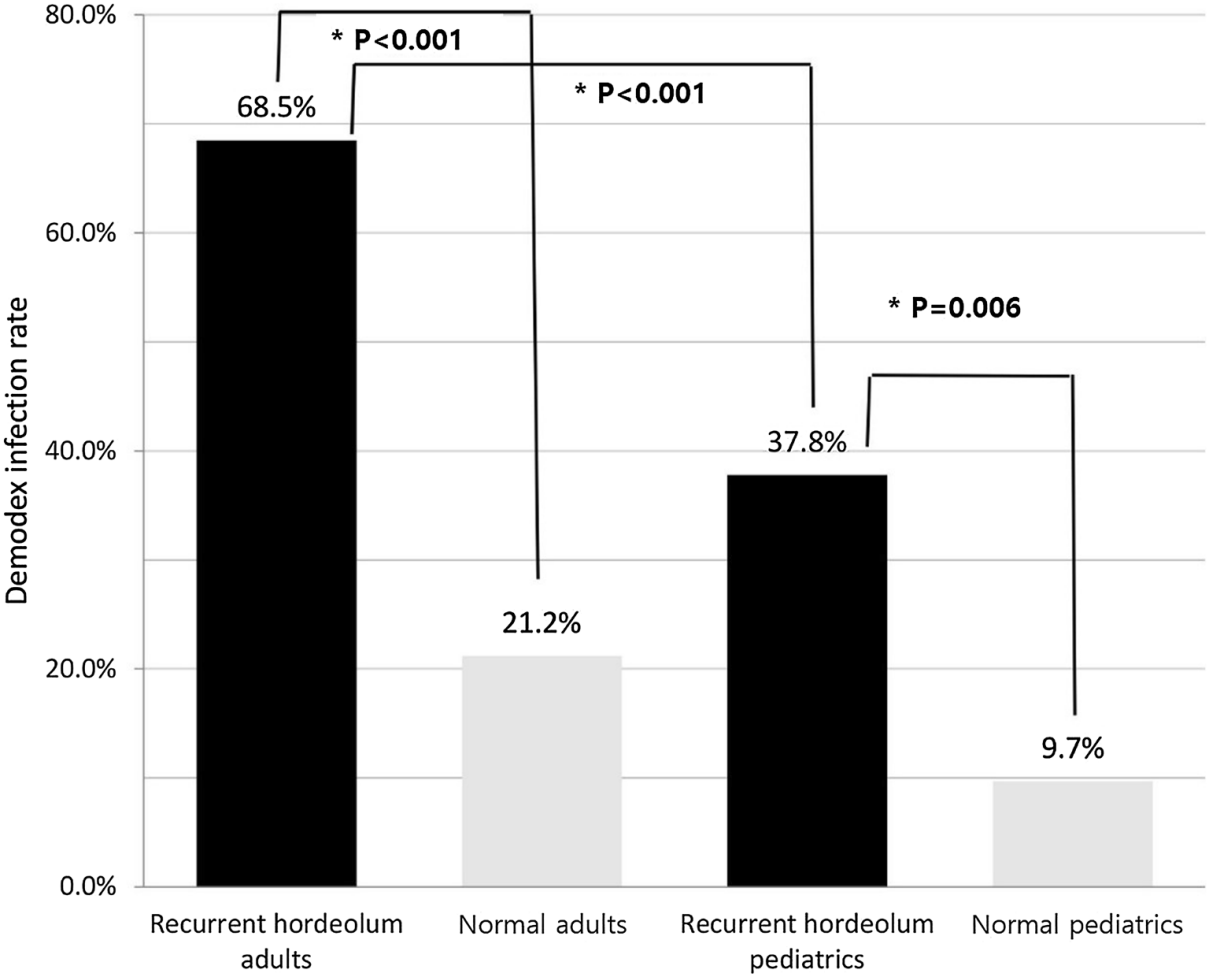
Incidence of *Demodex* infestation in adult and pediatric patients in the recurrent hordeolum and control groups. This graph demonstrates a significantly higher rate of *Demodex* infection among adult patients than among pediatric patients in the recurrent hordeolum group.

*Demodex* was detected in 91 (59.5%) and 17 (17.5%) patients in the recurrent hordeolum and control groups, respectively; the difference was statistically significant (*p* < 0.001) (Table 1). When the recurrent hordeolum group was analyzed according to age, *Demodex* was detected in 74 (68.5%) and 17 (37.8%) of the adult and pediatric patients, respectively; *Demodex* was significantly more prevalent in adult patients (*p* < 0.05). The number of mites was counted in 19 recurrent patients, and 1–15 mites were found in 15 patients (14 adults, 1 pediatric patient). In contrast, in the control group, *Demodex* was detected in 14 (21.2%) and 3 (9.7%) of the adult and pediatric patients, respectively. The rate of *Demodex* detection was significantly higher among adult patients than among pediatric patients in the recurrent hordeolum group than in the control group (*p*_*adult*_ < 0.001, *p*_*pediatric*_ = 0.006) (Fig. 3).

**Figure 4:**
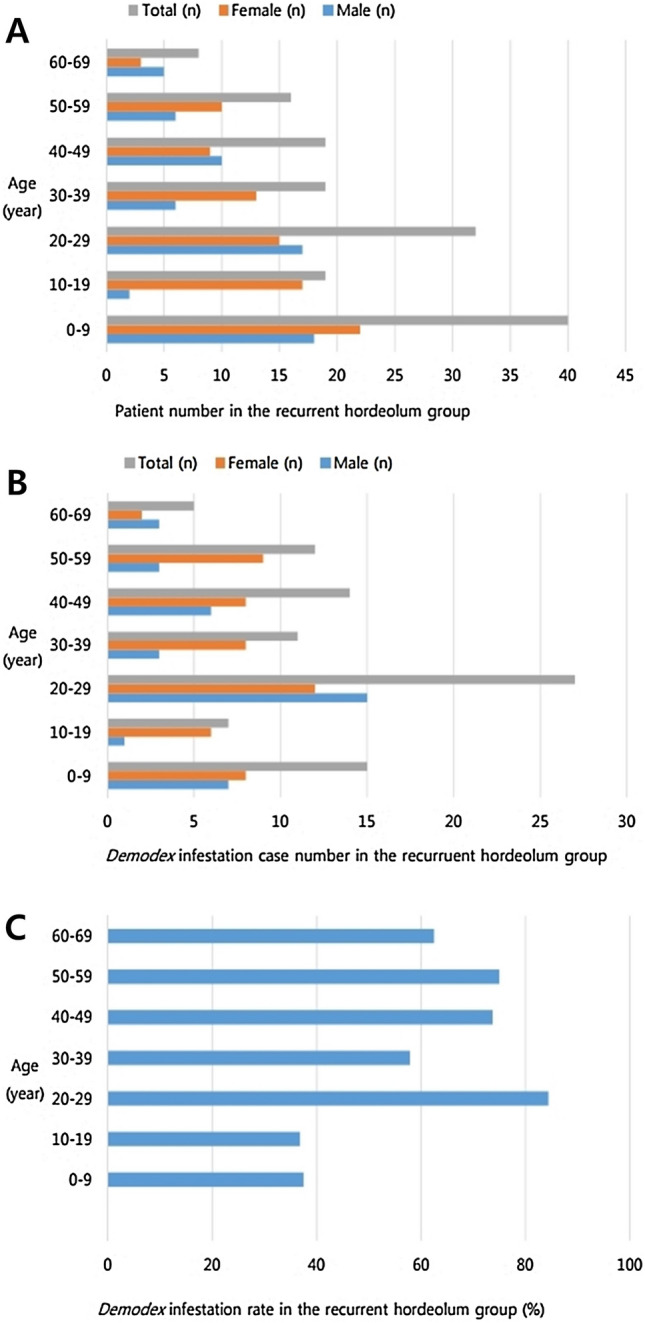
Age-specific distribution of recurrent hordeola and *Demodex* infestation. The graph demonstrates the highest frequency of hordeolum recurrence in patients aged < 10 years (**A**); however, patients in their 20s with recurrent hordeola had the highest case number of *Demodex* infestation (**B**) and the highest *Demodex* infestation rate (84.4%) (**C**) among the various age groups.

Recurrent hordeola were the most common in patients aged < 10 years, but *Demodex* mites were most frequently observed (84.4%) among patients in their 20s in the recurrent hordeolum group (Fig. 4). Among the patients with *Demodex* infestations, two-thirds of the recurrent eyelid lesions were characterized by focal inflammation of the lid margin such as external hordeola, whereas one-third were deep, roundish inflammatory lesions in the tarsal plate such as internal hordeola. The majority of patients (80.2%) in the *Demodex* group also had anterior blepharitis; anterior blepharitis was more common in the *Demodex* group than in the control group. Recurrent lesions were more likely to be located in the upper eyelid (57.1%) than in the lower eyelid (32.9%). Eyelids with *Demodex* infestations were significantly more likely to have more than one hordeolum than those without *Demodex* infestation (*p* = 0.008). Among adult patients with recurrent hordeola and *Demodex* infestations, the interval period between the primary incision and curettage and lesion recurrence was shorter (8.02 ± 5.42 weeks) than among patients without *Demodex* infestations (12.31 ± 7.05 weeks). In contrast, the interval period in pediatric patients, while also short, demonstrated no significant difference with or without *Demodex* infestation (Table 3). Our results suggested that *Demodex* infestation plays a more important role in hordeolum recurrence in adult eyelids than in pediatric eyelids.

**Table 3:** The interval period of recurrent hordeolum.

	Adult			Pediatric		
	*Demodex* (+)	*Demodex* (−)	*p*	*Demodex* (+)	*Demodex* (−)	*p*
Interval period (weeks)	8.02 ± 5.42	12.31 ± 7.05	0.004*	8.02 ± 5.42	13.53 ± 8.00	0.306

## Discussion

Internal and external hordeola are infections of the internal meibomian glands and external glands of Zeis and/or Moll, respectively, whereas chalazia are chronic lipogranulomatous inflammatory lesions that develop secondary to blockage of the meibomian gland orifices or stagnation of sebaceous secretions^8^. It is often challenging to distinguish between a chalazion and an internal hordeolum during the acute inflammatory phase of the chalazia^9^. Blepharitis, acne rosacea, trichiasis, and cicatricial ectropion have been found to be associated with internal hordeola^10–12^. In children, immunodeficiency, malnutrition, and vitamin A deficiency have been reported to be associated with chalazia^13,14^. A recurrent hordeolum usually occurs when the treatment fails to eliminate the bacteria that are present, completely and rarely is it due to a new infection^15^. Recurrent lesions should be assessed for the clinical features of malignancy, such as the presence of ulceration, telangiectatic vessels, or irregular or indistinct borders.

*Demodex* mites are normal inhabitants of human hair follicles and considered part of the normal flora of the pilosebaceous unit^1^. There are many species of *Demodex,* but only *Demodex folliculorum* and *D. brevis* are found in the human body^16^. *D. folliculorum* inhabits the eyelash hair follicles, whereas *D. brevis* lives deep within the meibomian glands^17^.

The prevalence of *Demodex* increases in older individuals, as well as in malnourished or immunosuppressed patients^18,19^. Several studies have shown that *Demodex* infestation is closely related to blepharitis; blepharitis has been identified as a major risk factor for chalazia^5,19,20^. Yam et al*.* demonstrated that 72.9% of adults with recurrent chalazia also had *Demodex* infestations^2^. Liang et al*.* reported that 69.2% of patients with chronic chalazia had *Demodex* infestations^3^. Schear et al*.* conducted a histopathologic study of eyelids with chalazia and reported that the average mite count per specimen was significantly higher than that in controls^21^. We found that *Demodex* was more prevalent among adult and pediatric patients (*p*_adult_ < 0.001, *p*_pediatric_ = 0.006) in the recurrent hordeolum group than in the control group (Table 1). This suggested that *Demodex* infestation has a potential pathogenic role in the development of recurrent hordeola in adult and pediatric patients.

Our study also found that recurrent eyelid lesions associated with *Demodex* infestations mostly showed external hordeolum (67%), while the rest looked like internal hordeola (23%) (Table 2). This means that *Demodex*-related lesions were more likely to involve the anterior lamella of the eyelid.

Recurrent hordeola developed after primary incision and curettage at shorter time intervals in the *Demodex* group than in the group without *Demodex* (Table 3), which indicated that *Demodex* infestation may be the most significant factor in the pathogenesis of hordeolum recurrence.

The pathophysiology of *Demodex*-associated eyelid lesions remains unclear; these lesions are believed to develop as an inflammatory foreign body granulomatous reaction to the chitinous exoskeleton of *Demodex* mites^22^. Mechanical obstruction of the meibomian glands results in a vicious cycle that contributes to *Demodex* overgrowth^2,3,20,23^. The *Demodex* mites also consume the epithelial cells of the eyelids, which induces micro-abrasions, epithelial hyperplasia, and reactive hyper-keratinization around the base of the lashes, forming cylindrical dandruff^17^. *Demodex* mites may also act as vectors for other pathogenic organisms that promote meibomian gland infection^3,23^. Overall, *Demodex* infestation can cause chronic blepharitis, conjunctival inflammation, and meibomian gland dysfunction. In addition, *Demodex* mites can penetrate the dermis and cause dermatological diseases, such as acne, rosacea, and folliculitis^24,25^.

*Demodex* infestation increases with age, and the mites are rarely found in newborns or children^19,26,27^. Arzola et al*.* reported that *Demodex* mites are observed in 64%, 75%, and 100% of patients belonging to 76–85-years, 86–95-years, and over 95-years of age groups, respectively. In contrast, only 11% of patients between 15 and 25-years of age demonstrated *Demodex* mites upon inspection^28^. In our study, the positivity rates for *Demodex* infestation in adult and pediatric patients with recurrent hordeola were 68.5% and 37.8%, respectively (Table 1). *Demodex* infestation in the recurrent hordeolum group was significantly more prevalent in adults than in children (*p* < 0.001) (Fig. 3). In the control group, the prevalence of *Demodex* showed no significant difference between adults and children (*p* = 0.123). Our data demonstrated that *Demodex* mites play a greater role in the development of recurrent lesions in adults than in children and may be due to an age-related difference in the *Demodex* infestation rate^26,27^. The maturity of the pilosebaceous unit may also be a factor^29–32^. Studies have reported that while sebum secretion in children is typically low, it reaches maximal rates at approximately 20-years of age^29–31^ and then, declines steadily, thereafter^32^. The low sebum secretion in children may provide poor environments for *Demodex* infestations. We also noted that the highest frequency of recurrent hordeola was observed in patients < 10 years of age (Fig. 4A); however, patients in their 20s with recurrent hordeola were the most likely group to have the highest concomitant *Demodex* infestation rate (84.4%) as well as the highest case number of *Demodex* infections among the overall enrolled recurrent hordeolum group (Fig. 4B,C). Moreover, the *Demodex* infestation rate in patients aged < 10 years with recurrent hordeola was 37.5% (Fig. 4C). These data suggest that the etiology of recurrent hordeola in the < 10 years age group could be related to factors other than *Demodex* infestation, and that the abundant sebum secretion rate which has been reported to be highest around 20 years of age^31^ or between 15 and 35 years of age^32^ may play an important role in Demodex survival in the 20s group.

TTO, Terpinen-4-ol, 4% pilocarpine gel, and systemic ivermectin have been advocated as the primary treatment for patients with *Demodex* blepharitis^6^. TTO is a natural essential oil that is steam-distilled from the leaf of *Melaleuca alternifolia*. It is an aboriginal, traditional, Australian medication that treats wounds and cutaneous infections and has antibacterial, antifungal, anti-inflammatory, and acaricidal properties^33^. Gao et al*.* evaluated the efficacy of various concentrations of TTO and other agents in eradicating *Demodex* mites^33–35^. Pure 100% concentrations of TTO, alcohol, dill weed oil, and caraway oil all demonstrated strong anti-*Demodex* effects, but these treatments had limited clinical use because of corneal toxicity and irritation. As such, lid scrub daily with tea tree shampoo and weekly application of 50% TTO is currently recommended for the eradication of ocular *Demodex* mites^33–35^. TTO is thought to work by washing out *Demodex* mites emerging from the cilia before they can mate and providing a sterilizing effect through its anti-cholinesterase activity^33,36^. Yam et al*.* noted that lid scrubs reduced chalazion recurrence by 96.8%^2^. In contrast, Koo et al*.* examined patients with *Demodex* blepharitis who were treated twice-daily with 10% TTO and once-weekly with 50% TTO lid scrubs in a clinical setting over 4 weeks. Their study reported a low *Demodex* eradication rate of 23.6%^7^.

This study has several limitations. First, we instilled a drop of saline on the epilated eyelashes when we mounted the specimens on the glass slide. This could have resulted in the underdiagnosis of *Demodex* infestations. Saline is also unable to dissolve cylindrical dandruffs, which can hide *Demodex* and allow them to remain in the follicle instead of taken along with the epilated eyelash. This can also reduce the total number of mites reported. Second, we did not analyze *Demodex* mites according to their species (*folliculorum* vs. *brevis*), which was done in previous studies, because we believed that identifying the species does not change the final treatment. Third, we did not count the mites in all study patients but only in 19 recurrent patients and found that there were 1–15 mites in 15 patients (14 adults, 1 pediatric patient). Therefore, while the cut-off level could not be exactly calculated between normal *Demodex* flora and demodicosis, we demonstrated a significant association between *Demodex* infestation and recurrent hordeola in the *Demodex* group compared to in the control group.

In this present study, we identified cases where the hordeola was associated with the clinical characteristics of external location, multiple numbers of lesions, or anterior blepharitis, without any signs of malignancy, but with recurrence within a short period of time despite the use of conservative treatment, and recommended that eyelash epilation be performed to identify the presence of *Demodex* mite. Alternately, eyelashes around recurrent hordeola should be observed carefully to identify *Demodex* migration or the presence of cylindrical dandruff at the base of eyelash because it is considered as the pathognomonic of *Demodex* infestation. In addition, we believe that further studies are required to determine the efficacy of eyelid cleansers for *Demodex*-associated recurrent eyelid lesions.
